# Integration of Teaching Processes and Learning Assessment in the Prefrontal Cortex during a Video Game Teaching–learning Task

**DOI:** 10.3389/fpsyg.2016.02052

**Published:** 2017-01-09

**Authors:** Naoyuki Takeuchi, Takayuki Mori, Yoshimi Suzukamo, Shin-Ichi Izumi

**Affiliations:** Department of Physical Medicine and Rehabilitation, Tohoku University Graduate School of MedicineSendai, Japan

**Keywords:** near-infrared spectroscopy, prefrontal cortex, metacognition, theory of mind, teaching

## Abstract

Human teaching is a social interaction that supports reciprocal and dynamical feedback between the teacher and the student. The prefrontal cortex (PFC) is a region of particular interest due to its demonstrated role in social interaction. In the present study, we evaluated the PFC activity simultaneously in two individuals playing the role of a teacher and student in a video game teaching–learning task. For that, we used two wearable near-infrared spectroscopy (NIRS) devices in order to elucidate the neural mechanisms underlying cognitive interactions between teachers and students. Fifteen teacher–student pairs in total (*N* = 30) participated in this study. Each *teacher* was instructed to teach the video game to their *student* partner, without speaking. The PFC activity was simultaneously evaluated in both participants using a wearable 16-channel NIRS system during the video game teaching–learning task. Two sessions, each including a triplet of a 30-s teaching–learning task, were performed in order to evaluate changes in PFC activity after advancement of teaching–learning state. Changes in the teachers’ left PFC activity between the first and second session positively correlated with those observed in students (*r* = 0.694, *p* = 0.004). Moreover, among teachers, multiple regression analysis revealed a correlation between the left PFC activity and the assessment gap between one’s own teaching and the student’s understanding (*β* = 0.649, *p* = 0.009). Activity in the left PFC changed synchronously in both teachers and students after advancement of the teaching–learning state. The left PFC of teachers may be involved in integrating information regarding one’s own teaching process and the student’s learning state. The present observations indicate that simultaneous recording and analysis of brain activity data during teacher–student interactions may be useful in the field of educational neuroscience.

## Introduction

Teaching is a type of human cognitive interaction during which the active communication between teacher and student results in the transmission of information or knowledge (Strauss et al., 2014). The relationship between a teacher and a student consists of multiple interrelated perceptions that both create from their interactions (Pianta et al., 2003). Moreover, in addition to the transmission of knowledge, teacher–student relationships have the power to significantly influence the student’s behavior. There is extensive evidence suggesting that the quality of teacher–student relationships is an important factor in a student’s competence in socio-emotional and behavioral functioning, as well as in academic skills (Pianta and Stuhlman, 2004; Buyse et al., 2008; Hughes et al., 2008; Zee et al., 2013). In the last decade, the field of educational neuroscience has gained recognition as advancements have been made in human cognitive neuroscience (Goswami and Szucs, 2011; Hruby, 2012; Sigman et al., 2014; Byrnes and Vu, 2015). However, the majority of the existing educational neuroscience studies have focused on the role of learning and development in students (Sigman et al., 2014; Byrnes and Vu, 2015). In order to elucidate the neural mechanism underlying teacher–student interactions, a neuroimaging method that allows simultaneous investigation in both teacher and student is required (Scholkmann et al., 2013; Liu and Pelowski, 2014). Speculating about the student’s behavior and learning state is a critical aspect of teaching and one of the main putative functional roles of the theory of mind (Watanabe, 2013; Strauss et al., 2014). In addition, teaching strategies depend upon the metacognitive process by which we monitor and control our own cognitive processes (Rodriguez, 2013; Shea et al., 2014; Kline, 2015). Thus, human teaching is an intentional interaction during which a teacher plans, monitors, and controls his or her own teaching strategy according to the student’s learning state. However, the between-individual neural mechanisms responsible for the integration of information in both parties are yet to be elucidated. Previous two-person neuroimaging studies of action imitation interactions mainly relied on body movement synchronization (Holper et al., 2012; Yun et al., 2012). However, tasks that involve rhythmic actions such as button pressing may also elicit rhythmic brain activities that could be misinterpreted as brain-to-brain interactions (Scholkmann et al., 2013). In order to combat this phenomenon, a recent study investigated the use of the Socratic method during debate interactions between a teacher and student, using functional near-infrared spectroscopy (NIRS) (Holper et al., 2013). In the present study, we investigated the neural mechanisms underlying teacher-student interactions by utilizing a video game task in order to minimize the involvement of rhythmic brain activity due to processes such as body movement synchronization. Furthermore, since video games can be taught via interaction with the character on the game screen, we excluded speaking in our teaching–learning game task in order to minimize the influence of brain areas associated with linguistic communication (Jiang et al., 2012, 2015).

In the present study, two wearable NIRS devices were used to simultaneously evaluate the prefrontal cortex (PFC) activity in two individuals during a video game teaching–learning task to elucidate the neural mechanisms underlying teacher–student interactions. The PFC is of particular interest due to its role in social cognitive interaction (Sanger et al., 2011; Scholkmann et al., 2013; Liu and Pelowski, 2014; Cheng et al., 2015). Therefore, we hypothesized that activity in the PFC in both teacher and student would change synchronously according to the teaching–learning state. To verify this hypothesis, we measured PFC activity during two teaching–learning sessions and evaluated changes in PFC activity after advancement of the teaching–learning state. Moreover, the PFC contributes to the complex mental states that underlie self-evaluative processing in cognitive control and theory of mind, which are both associated with comparing and integrating information about oneself and others (Raposo et al., 2011; Dixon et al., 2014). Considering the findings that teachers need the integration of information regarding their own teaching process and their students’ learning state (Rodriguez, 2013; Watanabe, 2013), we hypothesized that PFC activity in teachers may contribute to this integration process. To verify this hypothesis, we evaluated the correlation between PFC activity and assessments of teaching and learning. Reciprocal and dynamical feedback between teachers and students may be integral to successful teacher-student interactions through integration of information regarding the self and the others.

## Materials and Methods

### Participants

Thirty right-handed adults with no history of neurological abnormalities (15 pairs; 16 men and 14 women; mean age: 22.8 ± 2.9 years; range: 20–33 years) participated in this study. Participants were paired in same-gender dyads to avoid cross-gender effects (Balliet et al., 2011). Participants of each pair were randomly assigned to either the teacher or the student role during the video game teaching–learning task. The roles of each participant were maintained throughout the study. All participants provided written informed consent, and the protocol used in the study was approved by the local ethics committee of the Tohoku University Graduate School of Medicine (reference no. 2015-1-102).

### Video Game Task

**FIGURE 1:**
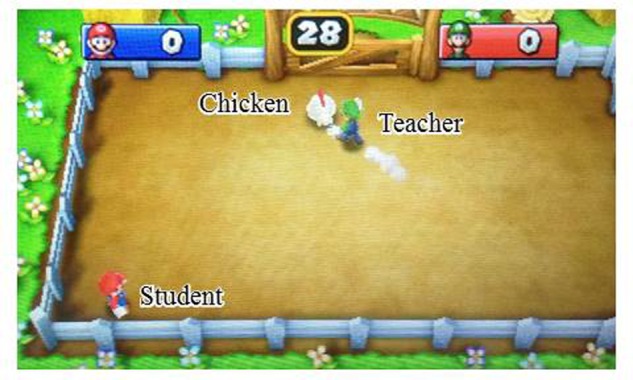
**Video game teaching–learning task.** In this game, players move their own characters on the game screen and hold a chicken to score as many game points. And they attempt to steal a chicken from one another by pushing a button when near another player. Teacher participants attempt to instruct student participants on how to play the game without speaking. This figure show a scene in which a teacher participant attempts to teach a student participant how to hold a chicken.

During the video game task, one participant (the teacher) is requested to teach the other (the student) how to play a mobile video game (Mario Party Island Tour, Nintendo Co. Ltd., Japan), while both participants are sitting side by side. We selected the mini game “Truckin’ and Cluckin”’ as the teaching–learning task (**Figure 1**). None of the participants had any prior experience with this game. In this mini game, players move their own characters and hold chickens to score as many game points as possible before time runs out. Players attempt to steal chickens from one another by pushing a button when close to the other player. The participants assigned to the teaching group (teachers) were given sufficient time to familiarize themselves with the game prior to the beginning of the experiment. In order to affirm that teachers had reached a consistent level of skill at the beginning of the teaching–learning session, this latter began only after teachers had beaten the computer for two consecutive times at the highest level of difficulty. Participants assigned to the student group (students) were allowed to familiarize themselves with moving the character prior to start of the experiment and were instructed to push the button only during the task session. Moreover, students were not instructed on how to attack other players. The role of the teachers was to instruct students, without speaking, regarding the following three points of the game: (1) having a chicken earns game points; (2) chickens can be stolen by attacking other players; and (3) players may be attacked even when they do not have chickens. We instructed the teachers to begin teaching about these after the student had successfully performed the aforementioned ascending order point twice. After teachers felt that they had sufficiently addressed all three points, they were instructed to allow students to play the game without interruptions. Each session began with a resting condition of approximately 30 s, followed by a task condition of 30 s, which was further followed by another rest condition of approximately 30 s. Participants completed three teaching–learning conditions per session. Participants were instructed to constantly face the game screen and to minimize head movements during all conditions. Sessions were performed twice, resulting in a total of six teaching–learning conditions.

### Teaching and Understanding Assessments and the Teaching–Understanding Gap

In order to evaluate the individual teaching and understanding assessments, participants were asked to report their own perceptions on teaching and understanding using a 100-point visual analog scale (0 points: not at all, 100 points: completely) following each task session according to these statements: (1) *teaching assessment* (teacher: I taught the student how to play this game; student: the teacher taught me how to play this game) and (2) *understanding assessment* (teacher: the student understood how to play this game; student: I understood how to play this game). The teaching–understanding gap was then calculated by subtracting the teaching assessment scores from the understanding assessment scores in each group in order to evaluate self–other distinctions.

### NIRS Measurement

**FIGURE 2:**
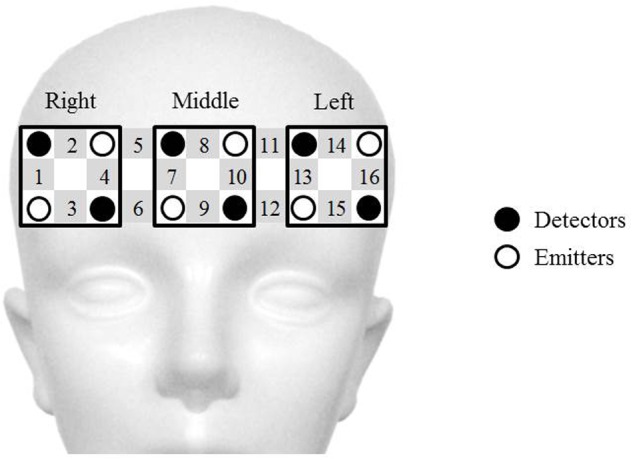
**Schematic representation of near-infrared spectroscopy (NIRS) probes and channels.** The NIRS system used in the present study consists of six emitters (white circles) and six detectors (black circles), resulting in 16 emitter-detector pairs, called channels (gray squares with channel numbers). The distance between the emitter and detector probe in each channel is set at 3.0 cm. Signals from the four channels over the right prefrontal cortex (PFC) (No. 1–4), middle PFC (No. 7–10), and left PFC (No. 13–16), respectively, are averaged.

Two wearable 16-channel NIRS systems (WOT, Hitachi Co. Ltd., Japan) were used to evaluate activity in the PFC while participants engaged in the video game teaching–learning task. A portable processing unit for controlling the optical topography measurements was connected to the probe unit through a flexible cable bundle. The processing unit sent data to a personal computer that controlled the experiment through a wireless local area network. **Figure 2** illustrates the NIRS probes and channels. The NIRS system used in this study consisted of six emitters and six detectors, resulting in sixteen channels, each consisting of one emitter-detector pair. In general, an emitter-detector distance around 2.5–3.5 cm is applied, because a distance below 2 cm might result in only superficial layer signal capture, while a distance above 4 cm might result in a weak signal (Gratton et al., 2006; Naseer et al., 2016a). In our study, this distance was set to 3.0 cm. The lowest probes were positioned along the Fp1-Fp2 line according to the international 10–20 system used in electroencephalography (Leon-Carrion et al., 2010). Changes in the concentrations of oxygenated (oxy) and deoxygenated (deoxy) hemoglobin (Hb) were calculated according to the absorbance change of 705-nm and 830-nm light in accordance with the modified Beer–Lambert law (Delpy et al., 1988; Maki et al., 1995). Changes in oxy-Hb values were used as indicators of changes in the regional cerebral blood volume, since oxy-Hb is more sensitive than deoxy-Hb is to changes associated with brain activation (Hoshi et al., 2001). The start of each session was manually marked on the NIRS data and corresponded to the time of the start sound produced by the game device at the beginning of each game round (Nintendo 3DS, Nintendo Co. Ltd., Japan).

### NIRS Data Analysis

The sampling frequency for the NIRS data was set at 5 Hz. We defined the NIRS analysis block as the period ranging from 20 s prior to the onset of the teaching–learning task to 10 s after task completion. Three blocks of data were obtained for each participant. A moving-average filter with a time window of 5 s and a band pass filter of low pass 0.2 Hz and high pass 0.01 Hz were used to remove slow drifts and high-frequency fluctuations. Data from the three analysis blocks were averaged for each participant. One drawback of the NIRS method is the variability of the path length, which is dependent on the structure of the scalp and superficial tissues over the brain (Haeussinger et al., 2011; Heinzel et al., 2013). To avoid this issue, the oxy-Hb data from each channel and for each participant were normalized using a linear transformation so that the mean ± standard deviation of the oxy-Hb levels in the 10–20 s time-window prior to the start of the teaching–learning task were 0 ± 1 (AU). This normalization was also useful for circumventing the influence of differential path-length factors between participants and between cortical regions (Harada et al., 2009). The NIRS data during the teaching–learning task were defined for statistical analysis as the mean of the data recorded during a 30-s period of the game teaching–learning task. To offset the low spatial resolution of NIRS and inter-individual anatomical variability, the four channels over the right PFC, the middle PFC, and the left PFC were averaged, respectively (**Figure 2**). We used the Platform for Optical Topography Analysis Tools (Hitachi Corporation, Japan) and MATLAB (MathWorks, Natick, MA, USA) software to analyze the NIRS data.

### Statistical Analysis

A two-way repeated-measures analysis of variance (ANOVA) was used to determine the effect of group (teacher vs. student) on each assessment parameter (teaching, understanding, and teaching–understanding gap) as a between-participants factor and the effect of period (first and second) as a within-participants factor. A three-way repeated-measures ANOVA was used to determine the effects of group on the NIRS data as a between-participants factor, and of site (right PFC, middle PFC, and left PFC) and period (first and second) as within-participants factors. A *post hoc* analysis was performed using Bonferroni’s correction to reduce the possibility of Type I errors. Possible correlations among the NIRS data between the teacher and student at each site were determined using the Pearson’s correlation coefficient test. Moreover, we evaluated the correlation of changes in NIRS from the first to the second session between teacher and student using Pearson’s correlation coefficient test. To evaluate the correlation of PFC activity with each assessment parameter (dependent variables), multiple regression analysis was performed using the NIRS data (right PFC, middle PFC, and left PFC) as an independent variable. Stepwise inclusion/exclusion of independent variables into the regression model was determined by *F* probability of *p* < 0.05 for inclusion and *p* > 0.1 for exclusion. Multiple regression analysis was performed separately for teacher and student groups according to the session, since differential changes in each assessment parameter were observed between the first and the second session for the teacher and student groups, as indicated in the Section “Results.”

## Results

### Teaching–Understanding Assessment

**FIGURE 3:**
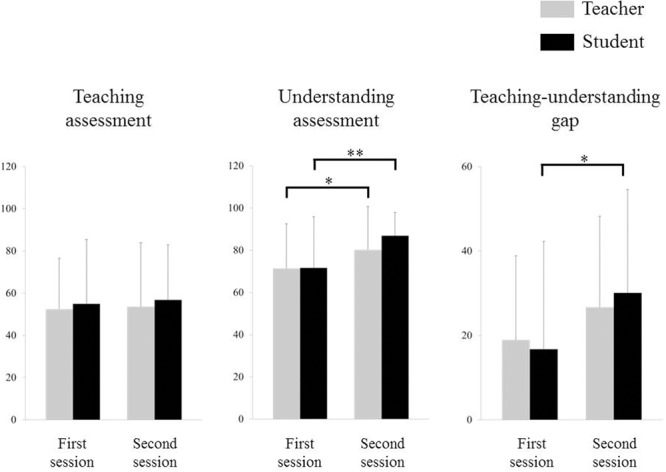
**Teaching–understanding assessments.** ^∗^*p* < 0.01, ^∗∗^*p* < 0.001, error bar: standard deviation.

**Figure 3** depicts the teaching–understanding assessment. Two-way repeated-measures ANOVA for the teaching assessment indicated no significant effect of period, group, or interaction effect between period and group. A two-way repeated-measures ANOVA for the understanding assessment, however, indicated a significant effect of period (*F*_1,28_ = 18.747, *p* < 0.001), but no significant effect of group or interaction effect between period and group. *Post hoc* testing revealed that ratings on the understanding assessment of the second session were higher than those obtained for the first session for both teachers (*p* = 0.004) and students (*p* < 0.001). A two-way repeated-measures ANOVA for the teaching–understanding gap indicated a significant effect of period (*F*_1,28_ = 7.445, *p* = 0.011), but no significant effect of group or interaction effect between period and group. *Post hoc* testing revealed that the teaching–understanding gap in the second session was larger than that in the first session in students (*p* = 0.002), but not in teachers.

### NIRS Data

**FIGURE 4:**
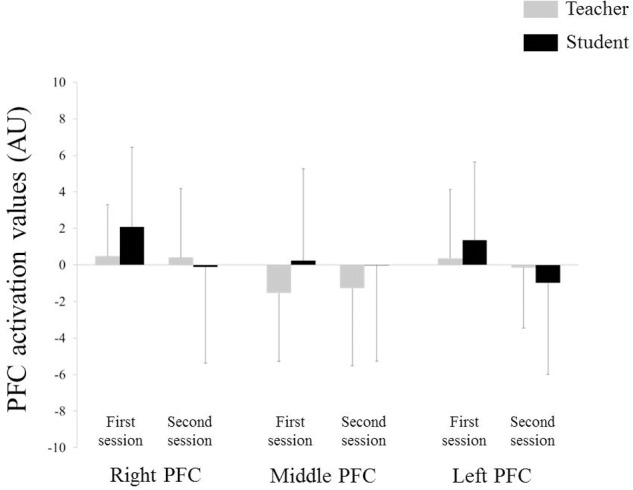
**Near-infrared spectroscopy (NIRS) data.** Error bar: standard deviation, PFC: prefrontal cortex.

**FIGURE 5:**
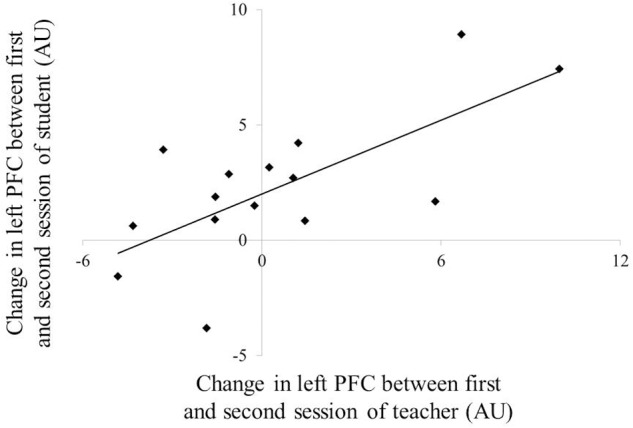
**Correlation of the change PFC activation between teacher and student.** PFC, prefrontal cortex.

**Figure 4** depicts the NIRS data during the teaching–learning task in both groups. Three-way repeated-measures ANOVA for NIRS values revealed no significant effect of group, site, or period, nor were there any statistically significant interactions. No correlation was observed in the PFC activity at neither of the sites between the teachers and students for each session. However, differences in left PFC activity between the first and second sessions were positively correlated between the teachers and students (**Figure 5**: *r* = 0.694, *p* = 0.004), but not in the middle or in the right PFC activity.

### Correlation of the PFC Activation With the Teaching–Understanding Assessment

**FIGURE 6:**
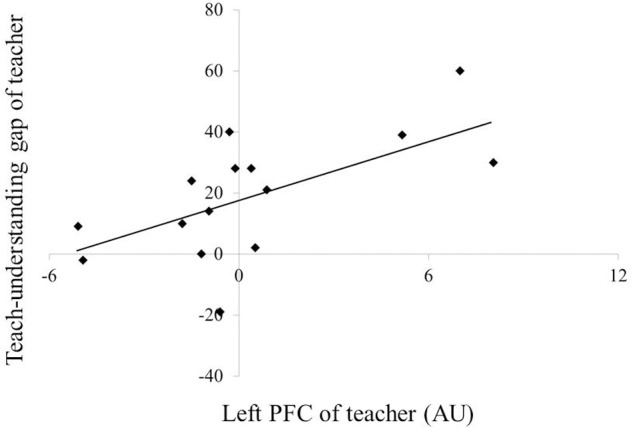
**Correlation of PFC activation with teach-understanding gap.** PFC, prefrontal cortex.

**Figure 6** depicts the correlations between PFC activation and teaching–understanding assessment scores. In teachers, multiple regression analysis revealed an association between the teaching–understanding gap and the left PFC activation during the first session (*R^2^* = 0.421, *F* = 9.445, *β* = 0.649, *p* = 0.009). However, no correlation between PFC activity and teaching–understanding assessment scores was observed in students.

## Discussion

The aim of the present study was to determine the extent to which PFC activity, as measured using NIRS, supports reciprocal, dynamical interaction between teachers and students during a teaching–learning video game task. Synchronous activity changes in the left PFC were observed in both teachers and students after advancement of the teaching–learning state. Moreover, left PFC activity in teachers was positively correlated with the assessment gap between one’s own teaching and the student’s understanding. These results indicate that the PFC plays an important role in non-verbal teaching–learning interactions.

### PFC Function during Teaching–Leaning Interaction

We observed synchronized activity changes in the left PFC in both teachers and students as the teaching–learning state advanced. As previously described, teaching is a dynamic phenomenon that changes according to the feedback given and information transferred between teacher and student (Watanabe, 2013; Strauss et al., 2014). The PFC is of particular interest since it plays a significant role in social interaction (Sanger et al., 2011; Scholkmann et al., 2013; Liu and Pelowski, 2014; Cheng et al., 2015). Therefore, the observed changes in the PFC activity for student–teacher pairs may represent a reduced demand for social interaction following successful teaching–learning tasks. This hypothesis is consistent with the results of previous two-person neuroimaging studies, which have reported synchronous hemodynamic activity of the PFC for student–teacher pairs who exhibited efficient communication (Holper et al., 2013). Additionally, the synchronous change of the PFC activity in both groups may be related to habituation to the game task and/or learning process, though not to the demand for social interaction. In fact, the understanding assessment in the second session scored higher than in the first session in both groups (teachers: *p* = 0.004, students: *p* < 0.001). It has been previously reported that an increase in activation takes place in the PFC during the learning process, which then decreases when the learning is complete (Leon-Carrion et al., 2010). Although there was no difference in PFC activity between the first and second session, the left PFC activity in each individual during the teaching–learning task may decrease after advancements have been made with respect to the learning state, especially in students.

Teachers must continually plan and monitor their own teaching process. This monitoring is associated with metacognitive functions, linking it closely with the concept of executive control (Frith, 2012; Rodriguez, 2013; Kline, 2015). Convergent evidence from a number of studies suggests a critical role for the rostrolateral PFC in metacognitive functions (McGaig et al., 2011; Fleming and Dolan, 2012). Therefore, we hypothesized that PFC activation in teachers would be related to the monitoring of their individual teaching processes. However, PFC activation in these participants correlated not with one’s own teaching assessment but with the assessment gap between one’s own teaching and the student’s understanding. Previous studies have reported that the PFC plays an important role in processes involved in comparing and integrating information about the self and the others (Raposo et al., 2011). Teachers need not only to plan and monitor their own teaching states but also to evaluate what their students know and what they are capable of doing (Rodriguez, 2013; Watanabe, 2013). These complex processes are critical when teachers must manage the information available regarding themselves and their students. Therefore, increased activity in the left PFC in the teaching group may reflect the integration of such information, as teachers attempt to adjust their own teaching strategies. However, the left PFC activation during the first session positively correlated with the teaching–understanding gap (*β* = 0.649, *p* = 0.009). This result indicated that the left PFC could have become activated even with low necessity to adjust the teaching strategy in the case when the student understood the game task well. These findings also indicate that the left PFC integrates the information regarding one’s own teaching process and the student’s learning state for monitoring rather than for adjusting the teaching strategy in the teaching–learning process.

### Limitations and Future Studies

We hypothesized that PFC activity in teachers may reflect the monitoring of teaching strategy and assessment of student’s learning state to facilitate the teaching–learning process. However, the results of the present study revealed activation in the PFC even when alterations to the teaching strategy were not required. Therefore, the role of the PFC in facilitating the teaching–learning process remains to be clarified. In order to directly confirm the possibility that activity in the PFC positively influences teaching–learning interactions, further studies are required to reveal whether changes in PFC activity following non-invasive brain stimulation would affect the teaching–learning process. Indeed, if the PFC activity were to facilitate the teaching–learning process, its feedback to the teacher during the teaching–learning task might act similarly as a brain–computer interface (BCI) neuro-feedback training. Recently, several studies had reported NIRS-based BCI focusing on the PFC activity (Naseer et al., 2016a,b). In order to evaluate whether the PFC signals during teaching–learning tasks are suitable for NIRS-based BCI training, it is a prerequisite to calculate the classification accuracies of pattern recognition and discrimination in BCI by cross-validation in future studies (Naseer et al., 2014). Moreover, the teaching–understanding gap in the second session was larger than that in the first session for the student group in our study. This observation indicates that students may have been able to learn the task on their own due to its simplicity. Future studies need to use other teaching–learning tasks with higher degree of difficulty and thus enhanced teaching necessity in order to fully evaluate the correlation between PFC activity and student–teacher interaction.

Although several studies have already reported a PFC activity in the right and/or left hemisphere during two-person interaction tasks (Funane et al., 2011; Cui et al., 2012; Holper et al., 2012, 2013; Cheng et al., 2015), the laterality of the PFC in social interaction remains to be clarified. Differences between tasks *per se* might partly contribute to the laterality of PFC activity observed during social interaction. Moreover, researchers have reported a positive association between the duration of using video games and cortical thickness in the left dorsolateral PFC (Kuhn et al., 2014). Therefore, the use of a video game teaching–learning task may have played a central role in our findings that teaching–understanding assessments exhibited a correlation with the left PFC activity. Future studies will require the use of tasks that are not based on video games in order to evaluate whether the laterality of PFC activity contributes to the teaching–learning interaction. In addition, future research should consider the involvement of other functional networks such as the human mirror neuron system, which plays an important role in two-person interactions (Iacoboni, 2009; Chatel-Goldman et al., 2013; Liu and Pelowski, 2014).

There are several limitations to be considered when interpreting the results of the present study. First, we need to consider the necessity for simultaneous acquisition of cerebral data from two persons in this study. Although the left PFC activity in both teachers and students changed in synchrony with the advancement of the teaching–learning state, we were unable to observe any correlation between teaching–learning assessment and PFC activity in students. These results indicate that the importance of simultaneous acquisition of brain activation between student and teacher in our study for revealing the neural mechanisms underlying teaching–learning processes may be relatively low. Therefore, future studies are required to elucidate the mechanisms underlying the teaching–leaning interaction from the student’s perspective. Another limitation of our study lies in the evaluation parameters. We relied only on subjective assessments using a visual analog scale to evaluate the teaching–learning state. Therefore, the objective parameters such as the game scores and eye tracking data must also be included to evaluate the teaching–learning performance in future studies. Finally, the lack of adjustment to the difficulty of the teaching–leaning task according to the participants may have resulted in the high variability observed in the NIRS data, masking thus differences in the PFC activity between the first and the second session. Moreover, individual anatomical differences may have influenced the observed variability in PFC activity. Indeed, the path length of the near infrared light and the NIRS sensitivity are both dependent on the scalp-to-cortex distance (Haeussinger et al., 2011; Heinzel et al., 2013). Although the NIRS data from each channel were normalized by linear transformation in each participant in order to circumvent these issues, future studies should address the impact of anatomical differences due to cortical, frontal sinus, and skull thickness using additional imaging methods (Haeussinger et al., 2011; Heinzel et al., 2013). Moreover, extra cortical physiological responses such as blood pressure, heart rate, and skin blood flow should be monitored to ascertain and account for the influence of these parameters on NIRS measurements (Kirilina et al., 2012; Erdogan et al., 2014).

## Conclusion

Activation of the left PFC in participants assigned to the teachers group may integrate information regarding the self and the others during non-verbal teaching–learning tasks in order to monitor the teaching process according to the students’ learning stage. The results of the present study support the notion that the role of the PFC in the teaching process is extremely complex, potentially indicating its involvement in the examination of both the learning state of the student and one’s own teaching state. Simultaneous investigation of neural activity in teacher–student interactions provides an opportunity to enhance our understanding of the dynamic process surrounding the flow of information between both parties. Our findings have the potential to impact further research in the field of human education.

## Author Contributions

NT conceived this study and was involved in conducting the experiments, processing data, and writing the manuscript. TM participated in data acquisition and analysis. YS participated in data analysis and interpretation. S-II participated in data interpretation and writing the manuscript.

## Conflict of Interest Statement

The authors declare that the research was conducted in the absence of any commercial or financial relationships that could be construed as a potential conflict of interest.
